# Concurrent fabry disease and immunoglobulin a nephropathy: a case report

**DOI:** 10.1186/s12882-023-03282-3

**Published:** 2023-11-01

**Authors:** Li-Na Zhou, Shao-Shao Dong, Sheng-Ze Zhang, Li-Wa Huang, Wen Huang

**Affiliations:** 1 Department of Nephrology, Wenzhou People’s Hospital, The Third Affiliated Hospital of Shanghai University, Wenzhou, 325000 Zhejiang Province China; 2 Department of Nephrology, The Second Affiliated Hospital of Wenzhou Medical University, No. 108 College Road, Wenzhou, 325000 Zhejiang Province China

**Keywords:** Left ventricular hypertrophy, Fabry disease, IgA nephropathy, Alpha-galactosidase A, Lyso-GL-3, *TTN* gene, *BAG*3 gene, Case report

## Abstract

**Background:**

Fabry disease (FD) is an X-linked, hereditary dysfunction of glycosphingolipid storage caused by mutations in the *GLA* gene encoding alpha-galactosidase A enzyme. In rare cases, FD may coexist with immunoglobulin A nephropathy (IgAN). We describe a case of concurrent FD, IgAN, and dilated cardiomyopathy-causing mutations in the *TTN* and *BAG*3 genes, which has not been reported previously.

**Case presentation:**

A 60-year-old female patient was admitted with a one-week history of facial and lower-limb edema, two-year history of left ventricular hypertrophy and sinus bradycardia, and recurring numbness and pain in three lateral digits with bilateral thenar muscle atrophy. Renal biopsy revealed concurrent FD (confirmed via an alpha-galactosidase A enzyme assay, Lyso-GL-3 quantification, and *GLA* gene sequencing) and IgAN. Heterozygous mutations in the *TTN* (c.30,484 C > A;p.P10162T) and *BAG*3 (c.88 A > G;p.I30V) genes were observed. The patient reported that two of her brothers had undergone kidney transplantation; one died suddenly at 60 years of age, and the other required a cardiac pacemaker. The 35-year-old son of the patient was screened for the *GLA* gene mutation and found to be positive for the same mutation as the patient. The patient was administered oral losartan (50 mg/day). Enzyme replacement therapy was refused due to financial reasons. Her renal and cardiac functions were stable yet worth closely monitoring during follow-up.

**Conclusion:**

The family history of patients with concurrent heart and renal diseases should be assessed in detail. Genetic testing and histological examinations are essential for diagnosing FD with IgAN.

## Background

Fabry disease (FD) [1, 2] is a rare, X-linked lysosomal storage disorder caused by mutations in the *GLA* gene that codes for the alpha-galactosidase A enzyme. The disease is characterized by the progressive accumulation of glycosphingolipids, including globotriaosylceramide (Gb3), in the lysosomes of the cells of multiple tissues and organs. The clinical manifestations of FD include angiokeratomas, hypohidrosis, neuropathic pain, corneal and lenticular opacities, gastrointestinal symptoms, cerebrovascular disease, and kidney and heart diseases. Progressive renal lesions [3] are a prominent feature of FD, and renal failure is a leading cause of late complications and mortality in patients with FD. Approximately 40% of patients with FD have left ventricular hypertrophy (LVH) at diagnosis [4]. However, it may be challenging to distinguish LVH from other heart diseases such as hypertension, valvular disease, hypertrophic cardiomyopathy (HCM), or infiltrative diseases such as amyloidosis.

Dilated cardiomyopathy (DCM) is a disease with a genetic background that is associated with mutations in genes encoding cytoskeletal, contractile, and other proteins. The *BAG3* (B cell lymphoma 2-associated anthanogene 3) gene codes for an anti-apoptotic protein on the sarcomere Z-disc. Mutations in *BAG3* are associated with DCM. Mutations in the gene encoding giant muscle filament titin (*TTN*) can also cause DCM.

This study reports a case of a 60-year-old female patient with a two-year history of LVH and sinus bradycardia. A renal biopsy revealed concurrent FD, immunoglobulin A nephropathy (IgAN), and DCM-causing mutations in the *TTN* and *BAG3* genes. To the best of our knowledge, this is the first report on such a case. Genetic testing and systematic histological examination are crucial in diagnosing this rare condition. The patient’s family history was also critical for diagnosing concurrent FD and IgAN.

## Case presentation

**Fig. 1:**
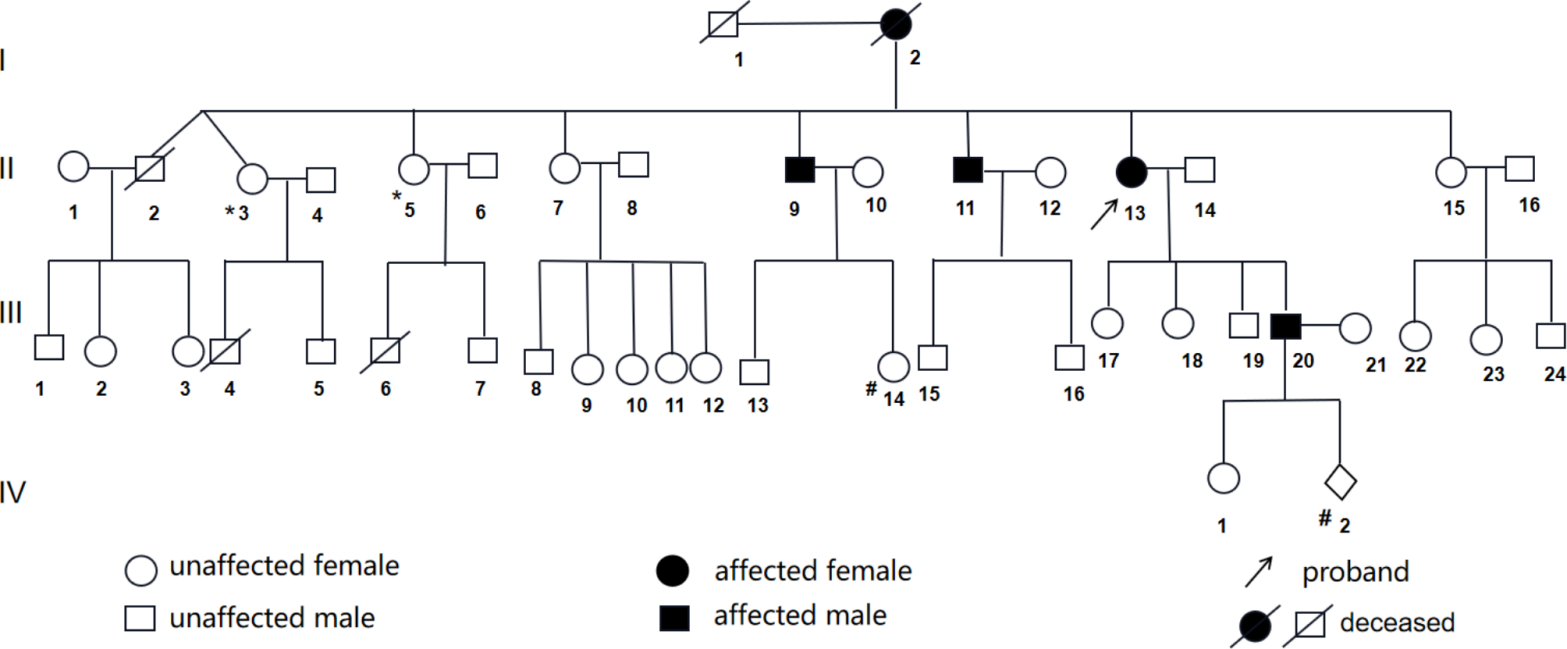
Patient’s family history. The patient’s mother (I2) died at 60 years of age, and her father (I1) died at 40 years of age, both for unknown reasons. The patient’s brother (II2) died suddenly at 60 years of age. Two other brothers of the patient required kidney transplants at 45 (II9) and 50 years of age (II11). One brother (II11) also required a cardiac pacemaker at 66 years of age. The patient’s sisters (II3 and II5) had minor microscopic hematuria. Two nephews of the patient (III4 and III6) died at 30 and 20 years of age, respectively, for unknown reasons. The patient’s niece (III14) had proteinuria, while the patient’s son (III20) had a 10-year history of proteinuria, bilateral finger angiokeratomas, and bilateral palm anhidrosis. He was diagnosed as having FD at 37 years of age. The fourth-generation offspring was a 3-year-old girl without any obvious symptoms at present and a 16 w + fetus with unknown sex

A 60-year-old female patient was admitted with a one-week history of facial and lower-limb edema, occasionally accompanied by slight chest tightness after walking. She had a two-year history of LVH and sinus bradycardia and a four-year history of numbness and pain in three of her lateral digits with bilateral thenar muscle atrophy previously diagnosed as cervical spondylosis. Upon review of her family history (Fig. 1), it was found that one of her brothers (II2) died suddenly at 60 years of age, whereas two other brothers underwent kidney transplantation at 45 (II9) and 50 years of age (II11). One patient (II11) required a cardiac pacemaker at 66 years of age. The patient had two sisters (II3 and II5) with a history of microscopic hematuria. The patient’s son (III20) had a 10-year history of proteinuria, angiokeratomas of the bilateral digits, and anhidrosis of the palms.

Upon admission, her body temperature was 36.5 ℃, and her blood pressure was 144/70 mmHg. Her blood pressure decreased to 125/65 mmHg on day 1 of hospitalization. Her respiratory rate was 20 beats/min, pulse rate was 41 beats/min, and no heart valve murmurs were observed. The patient had slight facial and bilateral lower-limb edema. Severe bilateral thenar muscle atrophy was also observed.

Urinalysis revealed no abnormalities, with a pH of 6.0, specific gravity of 1.015 (normal range, 1.003–1.030), no trace of protein, and no microscopic hematuria; 24-h urine protein was 1,254 mg (24-h urine volume was 2,524 mL), serum creatinine was 61 µmol/L, and the serum albumin level was 33.9 g/L. Indicators of cardiac function showed that creatine kinase, creatine kinase isokinase, and lactate dehydrogenase levels were slightly increased, while troponin I levels were within the normal range. Serum electrophoresis revealed decreased albumin and increased α1 globulin and γ globulin levels. At the same time, her free serum kappa-to-lambda ratio was 0.90 (kappa: 26.50 mg/L; lambda: 29.50 mg/L), which was in the normal range. Immunofixation electrophoresis revealed no monoclonal bands and no abnormalities in the bone marrow smears or biopsy. The remaining indicators, including blood routine index (leukocyte, hemoglobin, and platelets), hepatic enzymology (alanine aminotransferase and aspartate aminotransferase), electrolytes (potassium, sodium, chlorine, phosphorus, calcium, and magnesium), IgA, immunoglobulin E, immunoglobulin G, immunoglobulin M, Complement 3, Complement 4, C1q, antinuclear antibodies, anti-cyclic citrulline polypeptide antibody, anti-streptococcal hemolysin O antibody, rheumatoid factor, C-reactive protein, procalcitonin levels, and erythrocyte sedimentation rate, were all within normal limits (Table 1). No thyroid dysfunction was noted, and no tumors were detected on computed tomography or ultrasound throughout her body. No evidence of hepatitis B virus, hepatitis C virus, human immunodeficiency virus, or microspironema pallidum infections was observed.

**Table 1:** Patients’ Indicators

Items	Value	Normal range	Units
**Blood biochemical indicators**			
Serum creatinine	61	41–81	µmol/L
Urea	4.6	3.1–8.8	mmol/L
Uric acid	304.3	155–357	µmol/L
Serum albumin	33.9	40–55	g/L
ALT	36	7–40	U/L
AST	33	13–35	U/L
**Blood tests**			
Leukocyte	7.2	3.5–9.5	×10^9^/L
Hemoglobin	129	115–150	g/L
Platelet	252	125–350	x10^9^/L
**Electrolyte**			
Potassium	4.2	3.5–5.3	mmol/L
Sodium	147	137–147	mmol/L
Chlorine	111	99–110	mmol/L
Phosphorus	1.27	0.85–1.51	mmol/L
Calcium	2.13	2.11–2.52	mmol/L
Magnesium	0.83	0.75–1.02	mmol/L
**Cardiac function**			
Creatine kinase	145	30–135	U/L
Creatine kinase isokinase	5.74	< 3.77	ng/mL
Lactate dehydrogenase	309	120–246	U/L
Troponin I	0.019	< 0.120	ng/mL
**Electrophoresis**			
Albumin	51.1	53.8–62.2	%
α1 globulin	4.3	1.1–3.7	%
α2 globulin	11.1	7.4–12.6	%
β1 globulin	6.6	4.7–7.2	%
β2 globulin	6.2	3.2–6.5	%
γ globulin	20.7	9.2–18.2	%
**Immune-related indicators**			
IgE	29.2	< 100	IU/mL
IgA	2.92	1.0-4.2	g/L
IgG	13.6	8.6–17.4	g/L
IgM	0.57	0.5–2.8	g/L
C3	1.06	0.7–1.4	g/L
C4	0.29	0.1–0.4	g/L
C1q	161	159–233	mg/L
CRP	3.4	< 10	mg/L
RF	9.4	< 15.9	IU/mL
ASO	18	< 408	IU/mL
PCT	0.04	< 0.5	ng/ml
ESR	18	< 20	mm/h
SAA	11.4	0.0–10.0	mg/L
Serum β2-microglobulin	1.53	1.3-3.0	mg/L
**Coagulative fuction**			
PT	11.3	9.5–14.1	s
APTT	32.6	25.1–36.5	s
D-dimer	1.37	< 0.50	mg/L

**Abbreviations**: ALT = Alanine aminotransferase; AST = aspartate aminotransferase; IgE = immunoglobulin E; IgA = immunoglobulin A; IgG = immunoglobulin G; IgM = immunoglobulin M; C3 = complement 3; C4 = complement 4; CRP = C-reactive protein; RF = Rheumatoid factor; ASO = Anti-streptococcal hemolysin O antibody; PCT = Procalcitonin; ESR = Erythrocyte sedimentation rate; SAA = Serum amyloid A; PT = Prothrombin time; APTT = Activated partial thromboplastin time

**Fig. 2:**
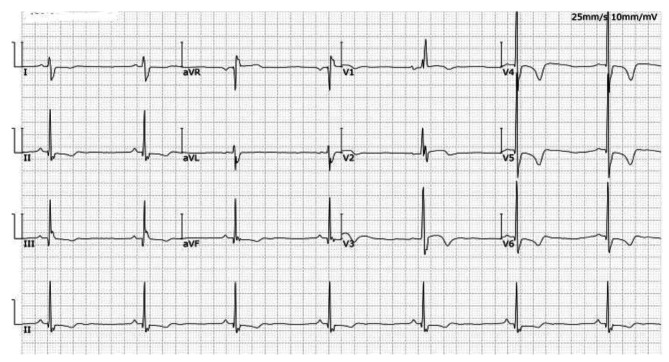
Electrocardiography results. Sinus bradycardia (41 beats/m), an incomplete right bundle branch block, and high voltage of the left ventricle were observed

**Fig. 3:**
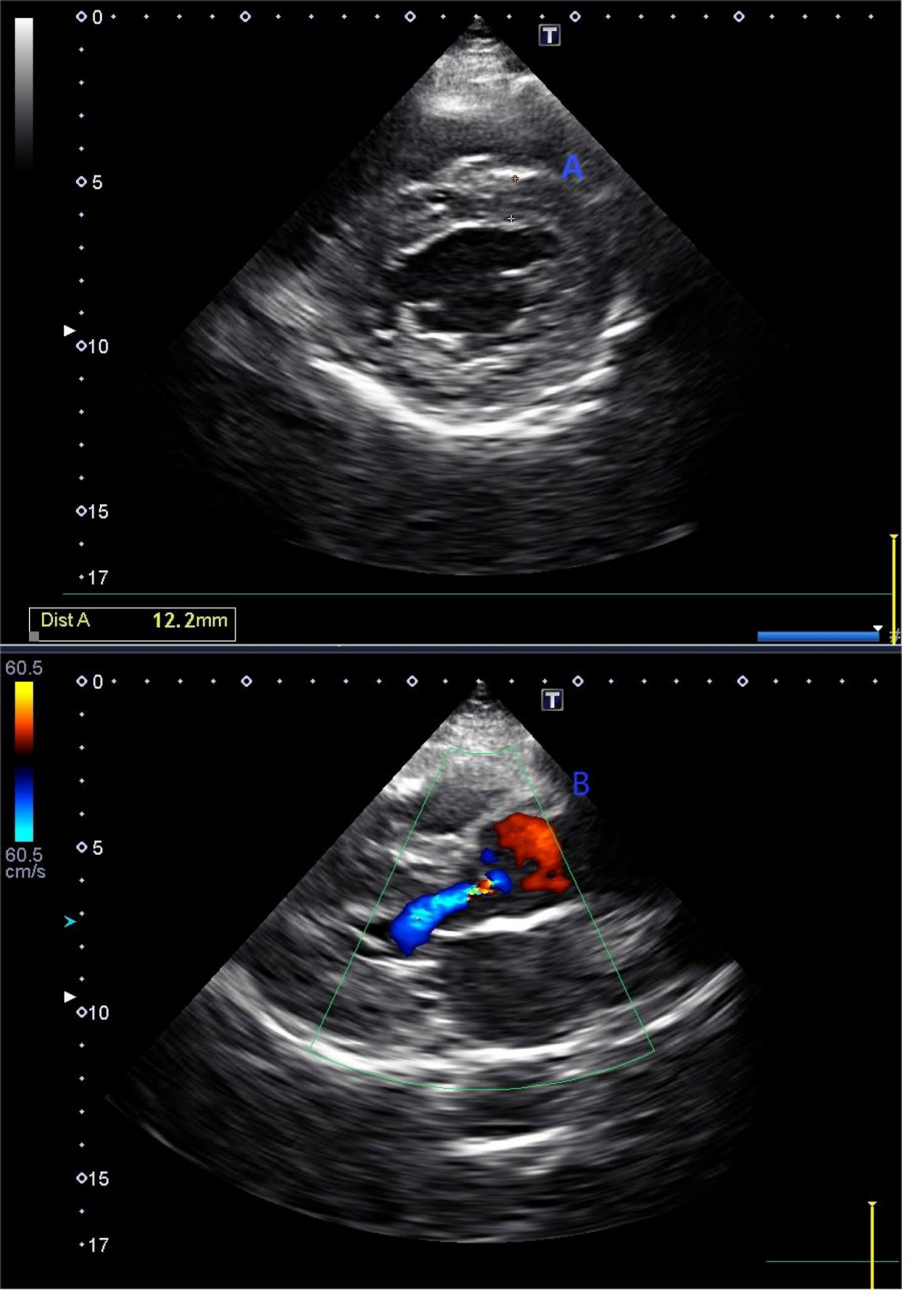
Echocardiography findings. Echocardiography revealed left ventricle wall thickening (**A**) and mild aortic valve regurgitation (**B**). The ascending aorta diameter was 30 mm, left atrium anteroposterior diameter was 34 mm, interventricular septum thickness in diastole was 12 mm, left ventricular end-diastolic dimension was 47 mm, left ventricular ejection fraction was 68%, left ventricular posterior wall thickness in diastole was 11 mm, left ventricular end-systolic dimension was 29 mm, main pulmonary artery was 21 mm, E was 0.85 m/s, A was 0.57 m/s, and E/A was 1.51

**Fig. 4:**
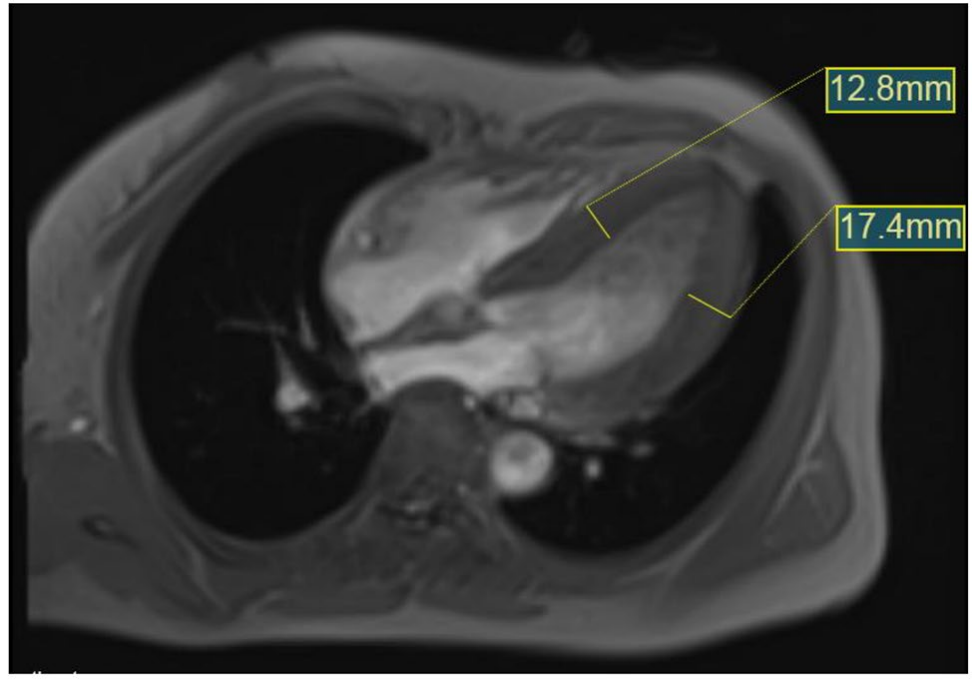
Cardiac magnetic resonance imaging results. The left ventricular septum and the left ventricular lateral wall in end-diastole are significantly thickened, with a maximum thickness of 17.4 mm. No significant enlargement of the left or right atrioventricular chambers and no expansion or stenosis of the pulmonary artery are observed

Electrocardiography revealed sinus bradycardia and LVH (Fig. 2). Echocardiography revealed left ventricular wall thickening and mild aortic valve regurgitation (Fig. 3). The left ventricular ejection fraction (LVEF) was 68%. Cardiac magnetic resonance (CMR) imaging showed a left ventricular wall thickness of 17.4 mm at end-diastole (Fig. 4).

**Fig. 5:**
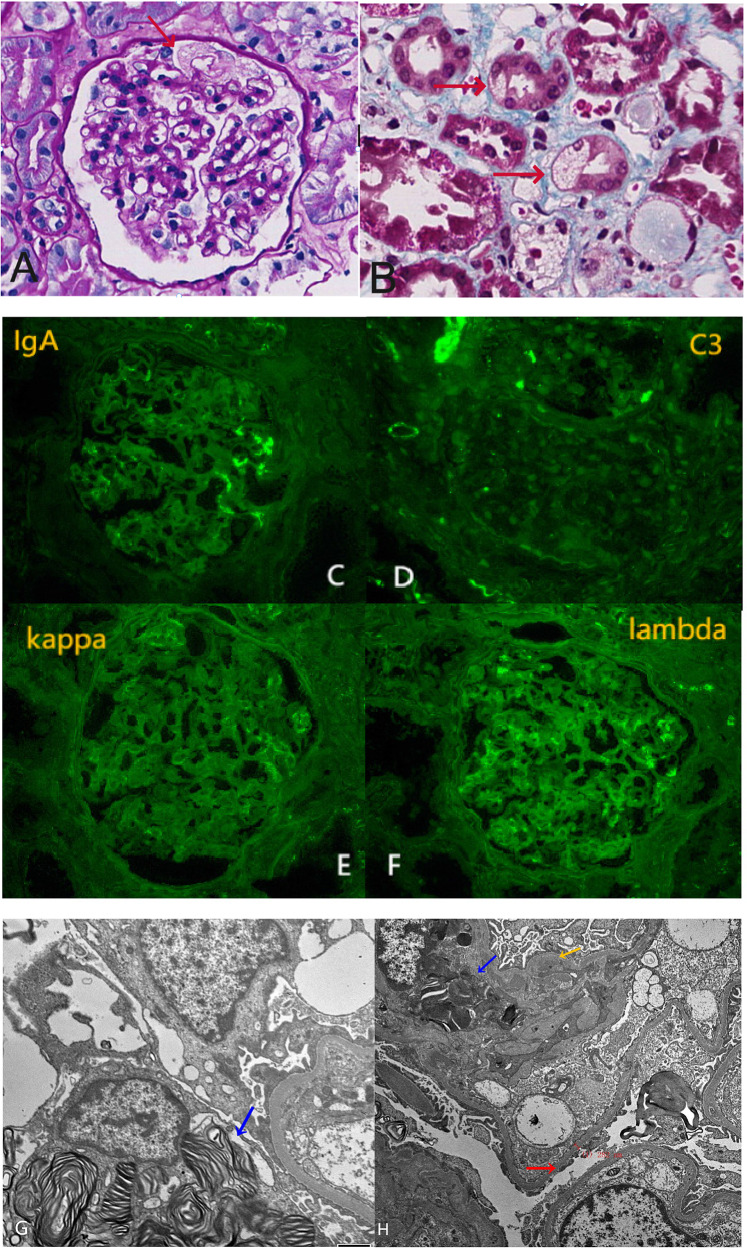
Renal biopsy pathology. **A**: (PAS ×400). **B**: (Masson ×400). Hyperplasic cells can be visualized in the glomerulus using light microscopy. Vacuolization of podocytes and renal tubular epithelial cells is shown (red arrow). **C, D, E, F**: Diffuse immunoglobulin A (++–+++), C3 (+), kappa (±–+), and lambda (++) segmental deposits in the mesangium are noted. G and H: Electron microscopy reveals myelin-like figures in the cytoplasm of podocytes (blue arrow), electron-dense deposits in the mesangium (yellow arrow), and partial foot process effacement (red arrow)

Renal biopsy revealed FD and IgAN (Fig. 5). PCR and gene sequencing identified *the GLA* gene mutation c.145 C > T (p.Arg 49 cys;chrX:100,662,747). The level of Lyso-GL-3 was 8.9 ng/mL(< 1.11), and that of alpha-galactosidase A enzyme was 1.85 µmol/L/h (2.40-17.65). A *BAG3* gene mutation (c.88 A > G (p.I30V;chr10:121411275)) and *a TTN* gene mutation (c.30,484 C > A (p.P10162T;chr2:179542423)) were also identified. The patient’s son was found to have the same *GLA* gene mutation (c.145 C > T) as the patient. His Lyso-GL-3 level was 58.15 ng/mL, and his alpha-galactosidase A enzyme level was 0.48 µmol/L/h.

**Fig. 6:**
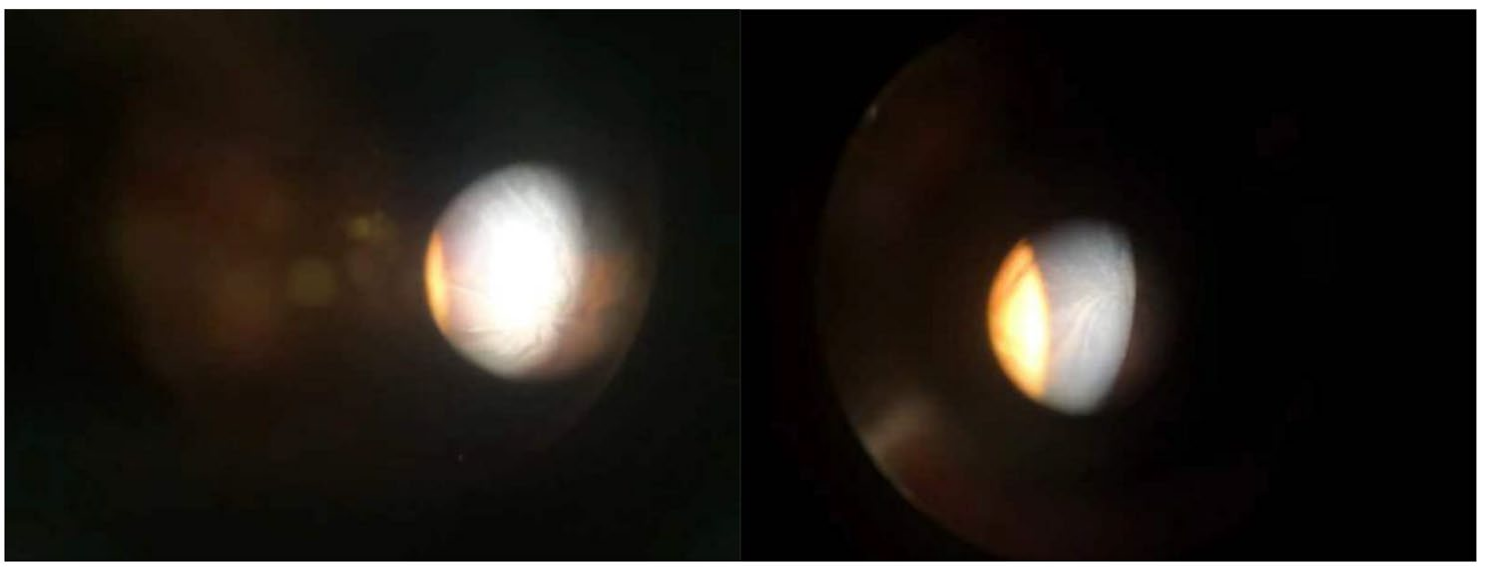
Ophthalmoscopy findings. No obvious abnormalities of the fundus are observed. A bilateral vortex-turbid cornea was observed

Electromyography revealed bilateral median nerve damage due to moderate-to-severe carpal tunnel syndrome. Ophthalmoscopy revealed no abnormalities in the fundus, although the corneas were vortex-turbid (Fig. 6). Computed tomography of the lungs and abdomen revealed mild bilateral pleural effusions, mild pericardial effusion, mild pelvic cavity effusion with coronary artery calcification, and an enlarged heart silhouette. No abnormalities were identified on ultrasound examination of the carotid artery, lower-limb arteries, or veins.

The patient was diagnosed with concurrent FD and IgAN. Losartan (50 mg/day) was administered orally. The patient refused enzyme replacement therapy (ERT) for financial reasons. Three months after discharge from the hospital, she developed a urinary tract infection that was cured with oral levofloxacin treatment. Her renal function remained stable after the infection was resolved, and her serum creatinine level was 65 µmol/L. Additionally, her cardiac function remained stable, with no chest tightness or other discomfort, and no deterioration of cardiac function was noted on cardiac ultrasound or other indicators. Her renal and cardiac functions were stable yet worth close monitoring during follow-up.

## Discussion and conclusions

Decreased enzyme activity and abnormally increased Gb3 or Lyso-Gl-3 levels are characteristic features of FD. Approximately 30% of female patients with FD have normal enzyme activities; therefore, genetic testing is crucial for its diagnosis. The *GLA* gene is located on chromosome Xq22.l, is 12 kb long, and consists of seven exons. A total of 674 mutations have been reported in this gene, of which approximately 60% are missense mutations [5]. A *GLA* mutation (c.145 C > T(R49C)) was observed in both the patient and her son. This mutation replaces the arginine at position 49 with cysteine. A previous study [6] reported the detection of this mutation in patients with FD and suggested that it may affect enzyme activity. Other variants, including Arg49Gly, Arg49Ser, Arg49Pro, Arg49Leu, and Arg49His, have been reported in patients with FD, suggesting that this locus may be a hotspot region.

*GLA* mutations that result in deficient enzymatic activity (< 5% of the normal mean) are associated with severe and early-onset phenotypes of FD [7]. In contrast, *GLA* mutations that lead to residual enzymatic activity are associated with attenuated and late-onset phenotypes of FD. Because this mutation occurs on the X chromosome, females are heterozygous carriers. The non-random inactivation mechanism of X staining [8] renders female alpha-galactosidase enzyme activity values within the normal range or only slightly decreased, which may account for differences in the clinical presentation between men and women [9]. Symptoms of FD occur earlier (typically in childhood) and are more severe in male patients, whereas female carriers often have milder and less prominent symptoms that appear in adulthood. However, heterozygote females exhibit a clinical spectrum that ranges from asymptomatic to disease as severe as that in affected males.

Significant variation was also observed between the genotypes and phenotypes of FD. The clinical manifestations of patients with FD with the same pathogenic gene mutation vary, and members of the same family may present with different clinical phenotypes. Phenotypic diversity was retrospectively observed in the pedigree of the patient. The patient experienced chronic neuropathic pain, undiagnosed LVH, and sinus bradycardia, whereas her son experienced proteinuria, anhidrosis, and angiokeratomas of the bilateral digits. The reasons for the phenotypic diversity in FD remain unclear. A previous study [10] reported monozygotic female twins with FD who presented with different phenotypes, suggesting that the clinical phenotype is determined by genetics, lifestyle, living environment, or polymorphisms in modifier genes.

Progressive renal lesions are central features of FD. Most hemizygous males with FD progress to end-stage renal disease at 30–40 years of age, while females may present with a range of renal involvement from asymptomatic to severe [11]. Renal symptoms, including proteinuria, microscopic hematuria, renal impairment, and decreased glomerular filtration rate, are the most common and important causes of late complications and mortality in patients with FD.

Fabry nephropathy is histologically characterized by the vacuolization of podocytes and epithelial cells under light microscopy. Lesions may also involve wall-layer epithelial, endothelial, mesangial, renal tubular epithelial, smooth muscle, and perivascular capillary endothelial cells. Blue bodies were observed in podocytes when toluidine blue staining was used on semi-thin sections, and myelin-like figures were observed by electron microscopy. In this patient, vacuolization was observed in podocytes and renal tubular epithelial cells under light microscopy, and myelin-like figures corresponding to Gb3 deposits were observed under electron microscopy. The patient had no history of amiodarone or hydroxychloroquine use [12], which is known to cause similar pathological changes.

Renal biopsy revealed diffuse glomerular mesangial cells and stroma slightly increased in light microscopy. Individual glomerular focal sac wall sclerosis or adhesion was also observed with occasional inflammatory cell infiltration. Individual renal tubular atrophy, scattered renal interstitial infiltration, and stromal focal mild fibrosis were noted. The Oxford classification of this case was M1E0S1T0C0. Diffuse immunoglobulin A (++~+++), C3 (+), kappa (± ~+), and lambda (++) segmental deposits in the mesangium were noted, and C4 and C1q were negative on immunofluorescence. Electron-dense deposits in the mesangium and myelin-like figures were observed in the cytoplasm of podocytes, suggesting concurrent IgAN and FD, which is a rare combination.

A previous study [13] reported that 80% of patients with concurrent IgAN and FD were from China and Japan, highlighting the relatively high prevalence of IgAN among ethnicities in East Asian countries. The average age of the patients was 34.5 years for men and 30.3 years for women, with no sex differences in the age of onset. It has been reported [14] that FD coexisting with IgAN mainly occurs in female patients, especially in end-stage renal disease. As the clinical course is rapid in male patients, symptoms generally appear during childhood. Therefore, the progression of FD to end-stage renal disease may precede the onset of IgAN in patients not subjected to renal biopsy. A report [15] of six patients (four men and two women) with concurrent FD and IgAN concluded that the pathological features are diverse and the clinical presentations are non-specific. Consequently, this rare coexistence may remain unrecognized or misdiagnosed. This issue is particularly important in female patients because of their asymptomatic clinical course. Therefore, the diagnosis of these patients relies on renal biopsy, and genetic diagnosis is needed to distinguish between IgA-nephropathy-complicated cases and uncomplicated FD.

Whether FD and IgAN share a common pathophysiology remains unclear. A previous study [16] suggested that the coexistence of FD and IgAN may be coincidental because the incidence of IgAN is high. However, Gb3 is structurally similar to a nephritogenic glycopeptide and may induce progressive glomerulonephritis [17]. Mesangial IgA levels disappeared after ERT in patients with FD, which may support the above rationale. The coexistence of FD and immune disorders such as systemic lupus erythematosus and rheumatoid arthritis has been described in previous studies. Antiphospholipid autoantibodies were detected in 45% of Argentine patients with FD, and antinuclear antibodies were detected in 39% of patients in an earlier study [18], suggesting a simultaneous autoimmune response, such as immune-mediated glomerulonephritis.

A previous study [19] reported that 40% of the patients with FD had LVH at the time of diagnosis. Conduction abnormalities, coronary artery disease, and valve lesions were also noted. Compared with that in controls, CMR imaging in patients with FD showed a significantly higher left ventricular mass (LVM) index, smaller left ventricular end-diastolic volume (LVEDV), higher LVEF, and lower stroke volume [20]. Except for the significantly increased thicknesses of the left ventricular septum and left ventricular lateral wall at end-diastole, CMR imaging showed no other characteristic findings of FD in our patient. Sinus bradycardia was observed in this patient. Cardiac dysfunction is caused by the accumulation of sphingolipids in cardiac myocytes and conduction tissues, which may explain the uniform ventricular wall thickness observed in patients with FD. However, distinguishing LVH from other heart diseases, such as hypertension, valvular disease, HCM, and infiltrative diseases, including amyloidosis, is challenging. Therefore, genetic testing is necessary to diagnose FD, particularly in patients with unexplained LVH.

Mutations in the *TTN* gene (c.30,484 C > A;p.P10162T) and heterozygous gene mutations in the *BAG3* gene (c.88 A > G,p.I30V) were detected in this patient. Because the patient’s parents were not screened, the source of these mutations was unclear. The functions of these genes are also undefined in relation to FD or IgAN. Previously reported mutant genes include *BAG3*, *TTN*, *LMNA*, *ACTC1*, *RBM20*, *MYH6*, and *MYH7*, and mutations in these genes have been associated with DCM. *BAG3* is an anti-apoptotic co-chaperone protein mutated in 2% of patients with DCM [21]. *BAG3* protects against DCM by reducing cardiomyocyte apoptosis, maintaining protein homeostasis, regulating mitochondrial stability, modulating myocardial contractions, and reducing cardiac arrhythmias. Some patients with FD present with DCM [22] and progress to the burnt-out phase, leading to hypertrophy and eccentric cardiac remodeling that results in a dilated, severely impaired left ventricle. Early-onset ventricular dilation was reported [23] in a 16-year-old patient with FD, suggesting that cardiac involvement can progress rapidly in some patients. To the best of our knowledge, this is the first report of concurrent FD and IgAN with these mutations in *TTN* and *BAG3* genes in a patient with LVH. However, the function of *TTN* and *BAG3* genes in our patient in terms of disease pathology remains unexplored. Approximately 16% of pathogenic DCM genes lead to HCM phenotypes, which may be related to the genotype. One limitation of our case report is the failure to perform a myocardial biopsy on this patient due to operational limitations. Therefore, we could not establish a cause-effect relationship between the genotype and phenotype, which requires further investigation. Thus, patients with such mutations should be followed up carefully.

ERT is the primary treatment for FD [24]. ERT with recombinant α-galactosidase A has been in use since 2001. There are two types of ERT, agalsidase alfa and agalsidase beta, each administered intravenously every other week at doses of 0.2 and 1 mg/kg, respectively. It has been demonstrated [25] that ERT may reduce or stabilize the LV mass and wall thickness, reduce the incidence of, and delay clinical events, while cardiac fibrosis is irreversible. In recent years, a novel oral pharmacological chaperone, Migalastat (Galafold®) [26], was approved in the European Union in 2016 and in the United States in 2018 for patients with FD, which increases the enzyme activity of “amenable” mutations. Compared with ERT, it showed similar results in terms of a reduction of LV mass, stabilization of kidney function, and plasma Lyso-Gb3. In summary, early treatment tended to result in better outcomes. However, the patient refused ERT due to financial reasons. Considering the relatively stable serum creatinine levels, oral losartan and traditional Chinese medicine were administered to reduce her urinary protein levels without steroid therapy. The renal and cardiac functions of the patient were stable yet still worth monitoring closely during follow-up.

In conclusion, cases of concurrent FD and IgAN are rare. Their clinical and pathological features are diverse. Patients with a family history of heart and kidney diseases should be closely monitored. Genetic testing and histological examination are essential to diagnose this rare condition. An accurate diagnosis will allow for early intervention with specific enzyme therapies.

## Data Availability

All generated or analyzed data in the presented case are included in this published article.

## List of abbreviations

*BAG3*: B cell lymphoma 2-associated anthanogene 3
CMR: cardiac magnetic resonance
DCM: dilated cardiomyopathy
ERT: enzyme replacement therapy
FD: Fabry disease
GLA: alpha-galactosidase A
Gb3: globotriaosylceramide
HCM: hypertrophic cardiomyopathy
IgA: immunoglobulin A
LVEF: left ventricular ejection fraction
LVH: left ventricular hypertrophy
*TTN*: titin

## References

[1] El-Abassi R, Singhal D, England JD (2014). Fabry’s disease. J Neurol Sci.

[2] Michaud M, Mauhin W, Belmatoug N, Garnotel R, Bedreddine N, Catros F (2020). When and how to diagnose fabry disease in clinical practice. Am J Med Sci.

[3] Fogo AB, Bostad L, Svarstad E, Cook WJ, Moll S, Barbey F (2010). Scoring system for renal pathology in fabry disease: report of the International Study Group of Fabry Nephropathy (ISGFN). Nephrol Dial Transplant.

[4] Kampmann C, Linhart A, Baehner F, Palecek T, Wiethoff CM, Miebach E (2008). Onset and progression of the Anderson-Fabry disease related cardiomyopathy. Int J Cardiol.

[5] Lukas J, Giese AK, Markoff A, Grittner U, Kolodny E, Mascher H (2013). Functional characterisation of alpha-galactosidase a mutations as a basis for a new classification system in fabry disease. PLOS Genet.

[6] Shin SH, Kluepfel-Stahl S, Cooney AM, Kaneski CR, Quirk JM, Schiffmann R (2008). Prediction of response of mutated alpha-galactosidase A to a pharmacological chaperone. Pharmacogenet Genomics.

[7] Arends M, Wanner C, Hughes D, Mehta A, Oder D, Watkinson OT (2017). Characterization of classical and nonclassical fabry disease: a multicenter study. J Am Soc Nephrol.

[8] Wagenhäuser L, Rickert V, Sommer C, Wanner C, Nordbeck P, Rost S (2022). X-chromosomal inactivation patterns in women with fabry disease. Mol Genet Genomic Med.

[9] Niemann M, Herrmann S, Hu K, Breunig F, Strotmann J, Beer M (2011). Differences in fabry cardiomyopathy between female and male patients: consequences for diagnostic assessment. JACC Cardiovasc Imaging.

[10] Gomez M, Molina L, Cladellas M, Ascoeta S, Soler C, Ble M (2012). Phenotype and genotype characterization and twin association in patients with Anderson-Fabry cardiomyopathy. Cardiology.

[11] Ramaswami U, Najafian B, Schieppati A, Mauer M, Bichet DG (2010). Assessment of renal pathology and dysfunction in children with fabry disease. Clin J Am Soc Nephrol.

[12] Sperati CJ, Rosenberg AZ (2018). Hydroxychloroquine-induced mimic of renal fabry disease. Kidney Int.

[13] Ren H, Li L, Yu J, Wu S, Zhou S, Zheng Y (2019). Fabry disease and immunoglobulin a nephropathy presenting with Alport syndrome-like findings: a case report. Med (Baltim).

[14] Kakita T, Nagatoya K, Mori T, Kobayashi M, Inoue T (2010). Coincidental finding of Fabry’s disease in a patient with IgA nephropathy. NDT Plus.

[15] Yang N, Wang X, Xu F, Zeng C, Wang J, Liu Z (2017). Clinical and pathological characteristics of fabry disease combined with IgA nephropathy in chinese patients. Clin Nephrol.

[16] Maixnerová D, Tesař V, Ryšavá R, Reiterová J, Poupětová H, Dvořáková L (2013). The coincidence of IgA nephropathy and fabry disease. BMC Nephrol.

[17] Shibata S, Takeda T, Natori Y (1988). The structure of nephritogenoside. A nephritogenic glycopeptide with alpha-N-glycosidic linkage. J Biol Chem.

[18] Navratil M, Ivkovic JI (2017). Chloroquine toxicity misdiagnosed as fabry disease associated with systemic lupus erythematosus and hashimoto thyroiditis. J Rheumatol.

[19] Yang X, Xie Y, Mi Y, Qinggang Li, Guangyan C, Xiangmei C. Clinical characteristics and relevant factors of cardiac and renal damage in patients with fabry disease. Chin J Jidney Dis Investig (Electronic Version). 2019; 2095-3216.2019.02.002.

[20] Kozor R, Grieve SM, Tchan MC, Callaghan F, Hamilton-Craig C, Denaro C (2016). Cardiac involvement in genotype-positive fabry disease patients assessed by cardiovascular MR. Heart.

[21] Norton N, Li D, Rieder MJ, Siegfried JD, Rampersaud E, Züchner S (2011). Genome-wide studies of copy number variation and exome sequencing identify rare variants in *BAG3* as a cause of dilated cardiomyopathy. Am J Hum Genet.

[22] Williams S, El-Medany A, Nightingale A, Ismail Y (2021). Rare presentation of fabry disease as ‘burnt-out’ hypertrophic cardiomyopathy. BMJ Case Rep.

[23] Tang J, Wu C, Cao J, Wang L (2020). Fabry disease with early-onset ventricular dilation: a case report. Med (Baltim).

[24] Lenders M, Brand E (2021). Fabry disease: the current treatment landscape. Drugs.

[25] Azevedo O, Cordeiro F, Gago MF, Miltenberger-Miltenyi G, Ferreira C, Sousa N, Cunha D (2021). Fabry Disease and the heart: a Comprehensive Review. Int J Mol Sci.

[26] Perretta F, Jaurretche S. Fabry Disease: switch from enzyme replacement therapy to oral chaperone migalastat: what do we know today? Healthcare (Basel). 2023; 11:449. 10.3390/healthcare11040449 PMC9957019 36832983

